# Acute Optimization of Squat Jump Performance in Trained Collegiate Sprinters through Knee Angle-Specific Isometric Training

**DOI:** 10.5114/jhk/208832

**Published:** 2025-11-20

**Authors:** Penglei Fan, Ting Wang, Youngsuk Kim, Zihao Zhao, Sukwon Kim

**Affiliations:** 1 College of Education and Sports Sciences, Yangtze University, Jingzhou, China.; 2 Institute of Student Physical Health Promotion, Jingchu University of Technology, Jingmen, China.; 3 Department of Physical Education, Jeonbuk National University, Jeonju, Republic of Korea.

**Keywords:** joint angle, stretching, conditioning, PAPE

## Abstract

This study investigated the activation of major lower limb muscles during isometric squats (ISs) at different knee angles and their effects as a conditioning activity (CA) on squat jump (SJ) performance at varying initial knee angles. Twelve collegiate sprinters were randomly assigned to three conditions: control (CC), 90°IS condition (90°ISC), and 120°IS condition (120°ISC), using a balanced crossover design. EMG data from five lower limb muscles were collected during ISs, alongside the peak center of mass velocity (V peak), peak force (F peak), peak power (P peak), average power (P avg), and the force-velocity ratio at peak power (SFV) during subsequent SJs. Results showed that the 90°IS condition elicited higher rectus femoris (p = 0.035, d = 0.729) activation, while the 120°IS condition led to greater soleus (p = 0.030, d = 0.759), and tibialis anterior (p = 0.022, d = 0.812) activation. Both the 90°ISC and the 120°ISC significantly increased peak velocity (90°ISC: p < 0.001, d = 1.379; 120°ISC: p < 0.001, d = 0.979) compared to the CC, but showed no significant improvements in F peak or P peak. Notably, the SFV during the 90° and 120°SJ was significantly lower in the 90°ISC and the 120°ISC compared to the CC (90°ISC: p < 0.001, d = −2.658; 120°ISC: p < 0.001, d = −1.785). These findings suggest that the IS at different knee angles selectively activates lower limb muscles and enhances velocity-related performance metrics. Specific IS angles can alter the force-velocity relationship, increasing the velocity component at peak power during subsequent movements. Coaches and athletes in speed-dependent sports are encouraged to select IS angles tailored to their specific training goals and performance needs.

## Introduction

The isometric squat (IS) can be used as a conditioning activity (CA) before an exercise task for a set period (Berning et al., 2010; Koźlenia et al., 2024). It is designed to temporarily enhance lower-limb muscle performance during subsequent exercise tasks, a phenomenon often defined as post-activation performance enhancement (PAPE) (Blazevich and Babault, 2019). The main physiological mechanisms underlying this enhanced muscle performance include the phosphorylation of regulatory myosin light chains, which increases the calcium sensitivity of contractile proteins and thereby facilitates cross-bridge formation between actin and myosin filaments (Blazevich and Babault, 2019). These changes alter the kinetics of myofiber activity, allowing the recruitment of higher-order motor units (Blazevich and Babault, 2019). Performance improvements are typically observed within three minutes of CA (Dobbs et al., 2019; Vargas-Molina et al., 2021) and peak after 7–10 minutes (Vargas-Molina et al., 2021; Wilson et al., 2013).

Previous studies have demonstrated that improvements in muscle performance are closely related to muscle fiber types, with fast-twitch fibers exhibiting more significant enhancements (Hamada et al., 2003). Furthermore, the magnitude of this performance enhancement appears to be modulated by the intensity and specificity of the conditioning activity. For instance, Esformes et al. (2013) reported that parallel squats, which involve greater knee flexion than quarter squats, elicited higher gluteal muscle activation and mechanical work output, ultimately resulting in superior performance improvements. There is evidence to show that the knee joint angle is a key determinant of force and torque generation (Krishnan et al., 2014; Tsoukos et al., 2016), and that variations in squat depth significantly alter muscle activation patterns (Barrett et al., 2023). Accordingly, IS performed at different knee joint angles are likely to produce varying levels of neuromuscular stimulation. Supporting this, Tsoukos et al. (2016) found that 90° ISs and 140° ISs had different effects on countermovement jump (CMJ) performance and suggested that isometric contractions at 90° ISs were more likely to induce muscle fatigue. This may be attributed to the different levels of stimulation applied to the lower limb muscles at varying knee joint angles during ISs, which in turn lead to differences in muscles performance enhancement.

Fatigue and performance enhancement occur simultaneously during CA (Tillin and Bishop, 2009; Tsoukos et al., 2016). Previous studies comparing three CA methods—concentric squats, eccentric squats, and ISs—revealed that ISs had the most significant effect on improving lower-limb performance in the CMJ (Bogdanis et al., 2014). Additionally, many researchers have suggested that, compared to concentric dynamic contractions, isometric contractions inherently have lower metabolic costs (Cady et al., 1989; Duchateau and Hainaut, 1984; Vargas-Molina et al., 2021) and are less likely to induce fatigue (Lum and Howatson, 2025) while enhancing subsequent explosive performance (Bogdanis et al., 2014; Vargas-Molina et al., 2021; Stastny et al., 2024). Numerous studies have also demonstrated the effectiveness of ISs in improving lower limb performance, particularly in jump height (Koźlenia et al., 2024; Krzysztofik et al., 2023; Tsoukos et al., 2016) and sprint speed (Krzysztofik et al., 2023; Lum et al., 2021). Knee joint angles of 90° and 120° are commonly used as starting positions for athletic movements (Harland and Steele, 1997; Li et al., 2024). However, it remains unclear whether ISs at these two angles provide different stimuli to the lower limb muscles, leading to distinct performance-enhancement patterns and improved movement performance by starting from different knee joint angles.

In this study, squat jumps (SJs) initiated at knee angles of 90° and 120° were used to evaluate the performance of athletes with varying knee joint starting angles. Investigating this issue will offer valuable guidance to athletes in selecting appropriate IS angles as a preparatory strategy tailored to the demands of specific sports and individual characteristics. This study aimed to explore the effects of ISs performed at different knee joint angles on the activation of major lower-limb muscles and their effect on lower-limb performance during SJs at different initial knee joint angles. Based on previous research, we hypothesized that ISs at different knee joint angles would provide distinct levels of stimulation to the lower limb muscles and, when used as a CA, would result in different power, force output, and velocity at different knee angles of the SJ, with a potential enhancement effect specific to each corresponding angle.

## Methods

### Experimental Design

These experiments used a balanced crossover and repeated-measures design in which all participants were randomly assigned to three different experimental conditions: a control condition (CC), a knee 90° IS condition (90° ISC), and a knee 120° IS condition (120° ISC). There was a 48-h interval between each experimental condition, during which no training or exercise was performed. Each experimental session was conducted between 15:00 and 17:00 (with meals occurring between 12:00 and 13:00), and participants were required to abstain from alcohol for 48 h and avoid caffeine for 8 h before testing. All participants performed a pre-test one week before the study commenced and were then randomly assigned to three conditions (CC, 90° ISC, and 120° ISC) to complete the main experimental sessions. Electromyography (EMG), force-time, and velocity-time curves, as well as peak power and mean power data, were collected from the lower limb muscles during 90° and 120° SJs.

### Participants

Twelve trained male collegiate sprinters were recruited for the study (age: 20.3 ± 0.9 years; body mass: 72.3 ± 8.1 kg; body height: 177.3 ± 6.6 cm; training experience: 5 ± 1.5 years; means ± SD). An *a priori* power analysis was conducted using G*Power 3.1.9.2 software (Heinrich Heine University Düsseldorf, Düsseldorf, Germany) for the formal experiment, which had a 3 × 2 (3 ISC and 2 SJ, within-subjects factors) experimental design, with an assumed effect size *f* = 0.25, an alpha level of 0.05, statistical power of 0.80, and a more conservative correlation among repeated measures of 0.55 selected based on pre-test data and a previous study (Mitchell et al., 2017). The analysis showed that a minimum sample size of 11 was sufficient. A larger sample size was selected to enhance statistical power and account for any unforeseen variability. Participants were required to have no history of lower extremity surgery, no injuries within the last six months, and maintain regular training routines > three times per week, each lasting > two hours, and refrain from using ergogenic supplements. Additional exclusion criteria included subjects who had a history of neurological disorders, significant cardiovascular issues, or musculoskeletal injuries that could affect lower limb performance. Written informed consent was obtained from each participant after a detailed explanation of the testing protocol, potential risks, and right to terminate participation at any time. All procedures followed the Declaration of Helsinki (1975), revised in 1996, and were approved by the Institutional Review Board of the Jeonbuk National University, Jeonju, Republic of Korea (protocol code: JBNU2022-04-008-002; approval date: 26 May 2022).

### Test Procedure

All participants underwent pre-experimental familiarization with the study’s procedures, and preliminary testing was conducted to ensure an understanding of the intervention and its procedures. During preliminary testing, ground reaction force (GRF) and EMG data were collected at knee angles of 90° IS and 120° IS. Post-test instructions and familiarization with the 90° SJ and 120° SJ movements were provided to minimize confounding factors during subsequent testing. At the start of the formal experiment, all participants completed a 5-min run on a treadmill at a self-selected pace as a warm-up. This was followed by 5 min of dynamic stretching focused on sagittal plane movements, including 2 sets of 10 walking lunges (5 per leg) over a distance of approximately 10 m, 2 sets of 10 walking toe touches (5 per leg), and 2 sets of 15 in situ air squats performed at a controlled tempo (~2 s per phase). A five-minute rest period was then included to stabilize body temperature and oxygen uptake before testing (Li et al., 2023). Afterwards, CA was performed, and five minutes after the end of CA, knee angles of 90° SJ and 120° SJ were tested to remain within the effect time of PAPE.

### Isometric Squat Interventions

The 90° ISC and 120° ISC used knee angles of 90° IS and 120° IS, respectively, as the CA, which was confirmed using an electrical goniometer (Biopac Systems, Inc., Goleta, CA, USA). All ISs were performed on a squat rack with a movable barbell positioned at the center, which was secured during the intervention to prevent any unintended knee joint movements while performing the IS (Loturco et al., 2024). Participants were first asked to perform an adaptive push under the barbell using 80% of their maximum force (based on pre-experimental data) as quickly as possible (Zabaloy et al., 2025). They were then asked to push under the barbell as fast as possible for 3 s at maximum force, completing 3 sets with a 1-min rest interval between each set. This type of conditioning activity has been used in many studies and is effective in improving lower-limb performance (Bogdanis et al., 2014; Tsoukos et al., 2016). Under the control condition, Participants performed three minutes of treadmill walking at a self-selected speed to maintain muscle temperature. Time measurements were recorded using a Casio HS-3V series stopwatch (Casio, Inc., Tokyo, Japan) to ensure accurate tracking of time intervals during the experiment.

### Squat Jump Test

All SJ tests were conducted on force plates with knee angles fixed at 90° and 120°, which were confirmed using an electrical goniometer. Before each jump, participants stood with their feet shoulder-width apart on two force plates, placed their hands on their waists, and maintained the specified knee angles for approximately 1 s in a squatting position. This setup was designed to eliminate the effects of arm swinging, stretch reflexes, and elastic muscle elements on jumping performance (Gillen et al., 2022). To avoid excessive downward movement, the drop in force at the start of the jump was not allowed to exceed five percent of the body weight. In the event that this threshold was exceeded, the jump was considered invalid. Participants were tested three times for each angle (90° SJ followed by 120° SJ), with a 60-s rest interval between each test and a 3-min rest interval between the two angles (Gillen et al., 2022; Li et al., 2023). This ensured that the tests occurred within the PAPE window after the intervention and minimized confounding factors (Li et al., 2023).

### Data Collection

EMG data were collected using a mobile EMG sensor (Trigno Avanti Sensor; Delsys, Natick, MA, USA). The skin was shaved and swabbed with alcohol swabs prior to placing the sensors (Fan et al., 2025). The placement locations were referenced to the Atlas of Muscle Innervation Zones (Barbero et al., 2012), and the sensors were aligned parallel to the muscle fibers to ensure accurate signal acquisition (Fan et al., 2025). EMG signals were recorded from the rectus femoris (RF), biceps femoris (BF), gastrocnemius lateralis (GL), soleus (SOL), and tibialis anterior (TA) muscles of the dominant leg at a sampling rate of 1,200 Hz. GRF data were acquired using force plates (Advanced Mechanical Technology, Inc., Watertown, MA, USA) at a sampling frequency of 1,200 Hz. Kinematic data were obtained using a Motive 2.2.0 (OptiTrack, Natural Point, Inc., Corvallis, OR, USA) motion capture system with 13 infrared cameras set at 120 Hz. Nineteen 14-mm diameter counter-view markers were placed on the participant's lower limbs for modeling using the CODA model. Kinematic, EMG, and GRF data were synchronized and collected using MOTIVE software.

### Data Processing

Pre-test EMG data were presented in Matlab 2021a (Math Works, Inc., Natick, MA, USA), band-pass filtered (20–450 Hz, Butterworth filter, 4^th^ order), and then full-wave rectified using low-pass filtering (6 Hz, Butterworth filter, 4^th^ order) to obtain a linear envelope (Fan et al., 2024). EMG data were normalized to the maximum value of each EMG channel to determine the EMG activation level (Fan et al., 2024). To assess the reliability of EMG measurements, the intraclass correlation coefficient (ICC) was computed using a two-way mixed-effects model with absolute agreement [ICC (3,1)], based on pre-test data collected across repeated trials. Confidence intervals (95% CI) for ICC values were also calculated. The two data sets with the highest ICC values (ICC > 0.90, 95%CI [0.91–0.98]) were selected, and their average was used for further analysis (Burden, 2010; Fan et al., 2024). For the pre-test, GRF data were filtered and smoothed using low-pass filtering (50 Hz, Butterworth filter, 4^th^ order) (Laughlin et al., 2011). Peak muscle activation and peak vertical GRF data were extracted at 90° and 120°IS during the pre-test. Initial analyses of the collected data were performed using Visual 3D software (C-Motion, Inc., Germantown, MD, USA). Skeletal models were built, and center of mass (COM) velocity data were calculated, defining the vertical GRF data five standard deviations above the static phase as the start of the take-off, and the first frame descending to less than 20 N as the take-off instant (Li et al., 2023; McLellan et al., 2011). The force and time data were filtered and smoothed using low-pass filtering (50 Hz, Butterworth filter, 4^th^ order) (Laughlin et al., 2011), whereas the center-of-mass velocity kinematic data were filtered and smoothed using low-pass filtering (16 Hz, Butterworth filter, 4^th^ order) (Fan et al., 2024). The filtered force, time, COM velocity, and time data were intercepted in stages and normalized to 101 data points (Li et al., 2023). Regarding force and velocity data from the three SJ tests, the two data sets with the highest consistency (ICC > 0.80, ICC [0.88–0.98]) were selected, and their mean values were used for further analysis (Janicijevic et al., 2021), and all force data were normalized to body weight. Power-time data were derived from the product of force and velocity (Li et al., 2023). Based on the force-time, velocity-time, and power-time data, the peak force (F peak), peak velocity (V peak), and peak power (P peak) were extracted, and the average power (P avg) was calculated. In addition, the ratio of force and velocity at the peak power moment (SFV), defined as the quotient of force and velocity at peak power, was calculated to describe the interrelationship between force and velocity at maximum power output (Samozino et al., 2012).

### Statistical Analysis

All statistical analyses were performed using SPSS v.25 (IBM Corp., Armonk, NY, USA). To compare lower-limb GRF and muscle activation at different IS angles, paired sample *t*-tests were conducted. To assess the effect of different IS and SJ angles on the outcome variables, a two-way repeated-measures analysis of variance (ANOVA) was performed, with IS and SJ angles as within-subject factors. The analysis examined the main effects of IS and SJ angles and their interaction. Data normality was tested using the Shapiro-Wilk test (*p* > 0.05) before this analysis. The Friedman’s test was used as a non-parametric alternative for data that did not conform to a normal distribution. When significant differences were found (*p* < 0.05), multiple comparisons were performed using Bonferroni’s *post hoc* correction. All data are presented as mean ± standard deviation (SD), with statistical significance set at α = 0.05. Effect sizes were interpreted as follows: for partial eta-squared (*η^2^*), values were categorized as trivial (< 0.01), small (0.01–0.06), moderate (0.06–0.14), and large effects (> 0.14); for Cohen’s *d*, values were categorized as trivial (< 0.20), small (0.20–0.49), moderate (0.5–0.79), and large effects (> 0.80) (Cohen, 1988).

## Results

### Pre-Test

A comparison between the 90° IS and the 120° IS showed that vertical peak GRF was significantly smaller for the 90° IS than for the 120° IS (*p* = 0.006, *d* = −1.039). A comparison of muscle activation levels between the 90°IS and the 120° IS revealed that the 90° IS had greater RF activation (*p* = 0.034, *d* = 0.729), but lower SOL (*p* = 0.030, *d* = 0.759) and TA (*p* = 0.022, *d* = −0.812) activation levels than the 120° IS.

### P Avg

The IS main effect had a significant impact on P avg (*p* = 0.003, *η^2^* = 0.328), while the interaction effect was not significant (*p* = 0.405, *η^2^* = 0.032). The main effect of the SJ was significant (*p* = 0.010, *η^2^* = 0.264). *Post hoc* analyses revealed that P avg was higher after the 120° ISC than after the CC (*p* = 0.010, *d* = 0.951) and the 90° ISC *(p* = 0.016, *d* = 0.759). Additionally, P avg was lower at the 90° SJ than at the 120° SJ (*p* = 0.010, *d* = 0.813).

### P Peak

P peak showed no significant differences for the IS main effect (*p* = 0.579, *η^2^* = 0.014), the interaction effect between the IS and the SJ (*p* = 0.647, *η^2^* = 0.01), or the SJ main effect (*p* = 0.27, *η^2^* = 0.055).

### F Peak

The IS main effect did not significantly affect the F peak (*p* = 0.619, *η^2^* = 0.011), but the interaction effect showed a significant difference (*p* = 0.026, *η^2^* = 0.206). In addition, the main effect of the SJ on the F peak was highly significant (*p* = 0.001, *η^2^* = 0.446). Simple effects analysis showed that the F peak was significantly higher in the 120° ISC than in the 90° ISC during the 90° SJ (*p* = 0.004, *d* = 1.080). When performing the 120° SJ, the F peak was lower at the 90° ISC than in the CC (*p* < 0.001, *d* = −1.407) and the 120° ISC (*p* = 0.002, *d* = −1.125). Additionally, the F peak at the 90° SJ was significantly lower than that at the 120° SJ in all groups (*p* < 0.001).

### V Peak

The IS main effect on V peak was significant (*p* = 0.001, *η^2^* = 0.603), but the interaction effect (*p* = 0.150, *η^2^* = 0.092) and the SJ main effect (*p* = 0.777, *η^2^* = 0.063) were not. *Post hoc* analyses indicated that the V peak was significantly higher for both the 90° ISC (*p* < 0.001, *d* = 1.379) and the 120° ISC (*p* < 0.001, *d* = 0.979) than for the CC.

### SFV

The IS main effect was significant for the SFV (*p* = 0.003, *η^2^* = 0.331), as was the interaction effect (*p* = 0.002, *η^2^* = 0.356), while the SJ main effect was highly significant (*p* < 0.001, *η^2^* = 0.999). Further simple effect analyses showed that during the 90° SJ, the SFV was lower in the 90° ISC compared to the CC (*p* < 0.001, *d* = −2.658) and the 120° ISC (*p* < 0.001, *d* = −2.080). At 120° SJ, the SFV in the 120° ISC was lower than that in the CC (*p* < 0.001, *d* = −1.785), and the SFV in the 90° ISC was also lower *(p* = 0.017, *d* = −0.759). Additionally, the SFV during the 90° SJ was lower (*p* < 0.001) than that during the 120° SJ in both the CC and the 90° ISC, whereas no significant difference was observed in the 120° ISC.

**Table 1:** Pre-test vertical GRF and muscle activation comparison results, shown as mean ± SD.

		90° IS	120° IS	T-test (*p*)
GRF (N • N^−1^)	Vertical GRF	2.639 ± 0.277	3.658 ± 0.904	0.007 **
Muscle	RF	0.960 ± 0.062	0.735 ± 0.197	0.036 *
	BF	0.980 ± 0.040	0.917 ± 0.084	0.146
	GL	0.719 ± 0.234	0.949 ± 0.079	0.062
	SOL	0.745 ± 0.192	0.969 ± 0.076	0.030 *
	TA	0.760 ± 0.192	0.979 ± 0.040	0.023 *

Note: 90° IS: knee 90° isometric squat; 120° IS: knee 120° isometric squat; GRF: ground reaction force; RF: rectus femoris; BF: biceps femoris; GL: gastrocnemius lateralis; SOL: soleus; TA: tibialis anterior; * is significantly different (p < 0.05); ** is very significantly different (p < 0.01)

**Figure 1:**
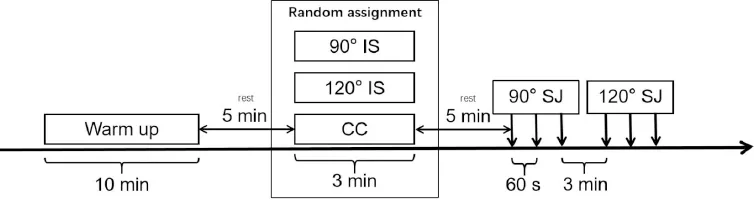
Schematic diagram of the experimental flow. CC: control condition; 90° IS: knee 90° isometric squat; 120° IS: knee 120° isometric squat; 120° SJ: 120° squat jump; 90° SJ: 90° squat jump

**Figure 2:**
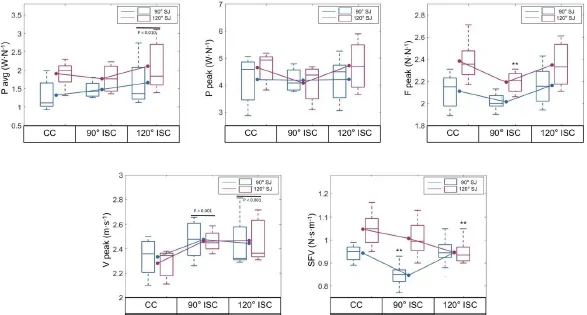
Box plots and line plots of the main variables associated with lower limb performance at the 90°SJ and 120°SJ for the CC, the 90°ISC, and the 120°ISC. CC: control condition; 90° ISC: knee 90° isometric squat condition; 120° ISC: knee 120° isometric squat condition; 120° SJ: 120° squat jump; 90° SJ: 90° squat jump; P avg: average power; P peak: peak power; F peak: peak force; V peak: peak velocity; SFV: the ratio of force and velocity at the peak power moment; is a difference in the 90° ISC and the 120° ISC compared to the CC; * is a significant difference (p < 0.05) in the 90° ISC and the 120° ISC at the 90° SJ or the 120° SJ compared to the CC; ** is a very significant difference (p < 0.01) in the 90° ISC and the 120° ISC at the 90° SJ or the 120° SJ compared to the CC

## Discussion

The findings of this study can be summarized as follows. First, the pre-test results indicated that RF activation was greater during the 90° IS, while SOL and TA activation was greater during the 120° IS, suggesting that the 90° IS and the 120° IS elicit different levels of muscle stimulation. Second, neither the 90° ISC nor the 120° ISC showed a higher F peak or P peak during the SJ than during the CC; however, a higher V peak was observed, and the 120° ISC showed a higher P avg than during the CC. Third, the 90° ISC showed a lower SFV at the 90° SJ, with no significant change at the 120° SJ when compared to the 120° ISC and the CC. Similarly, the 120° ISC exhibited a lower SFV at the 120° SJ, with no significant change at the 90° SJ. These findings support our hypotheses.

Numerous studies have demonstrated that the IS can enhance lower limb performance. Koźlenia et al. (2024) found that after performing three sets of 4-s IS exercises at 70% RM with knee joint angles < 90°, trained athletes experienced significant improvements in lower limb explosive power. Research by Krzysztofik et al. (2023) showed that the maximum IS at a 90° knee angle significantly enhanced jump height and sprint speed, with greater improvements observed in sprint speed than in vertical jumps following the conditioning activity (Seitz and Haff, 2016). It is important to note that Krzysztofik et al. (2023) used a different IS protocol (3 sets of 3 repetitions per set, with 3-min rest intervals between sets and 27-s isometric contraction duration) compared to the 9- s IS protocol employed in this study, which limits direct comparison between the studies. Similarly, in the present study, we observed an increase in the V peak after the IS at both angles. This can be attributed to the IS-induced PAPE effect, which involves the phosphorylation of myosin light chains. This mechanism enhances calcium ion sensitivity, optimizes neuromuscular efficiency, accelerates neuromuscular responses, and improves muscle recruitment efficiency (Blazevich and Babault, 2019; French et al., 2003; Vargas-Molina et al., 2021). However, performance improvements induced by ISs at different knee angles vary (Tsoukos et al., 2016). This may be because the degree of muscle potentiation is proportional to the stimulus intensity within a certain range (Esformes et al., 2013). These results suggest that the IS at a 90° knee angle primarily stimulates the thigh muscles, whereas the IS at a 120° angle predominantly activates the calf muscles. These differences may lead to distinct enhancement patterns in lower-limb performance, resulting in varying degrees of performance improvement depending on the knee angle used during the IS.

The performance enhancement induced by PAPE often occurs concurrently with fatigue. Tsoukos et al. (2016) demonstrated that compared to a 90° IS, a 140° IS was more effective in improving lower-limb performance. However, in athletes with superior jumping ability, the 90° IS significantly enhanced CMJ performance (Tsoukos et al., 2016). This can be attributed to their greater physical strength and a higher proportion of fast-twitch muscle fibers, which enables the activation of more muscle fibers and consequently, better performance (Hamada et al., 2003; Tsoukos et al., 2016). Additionally, research has indicated that muscles tend to fatigue more readily when working for longer periods (Smith et al., 2011), and strength gains from isometric contractions are often inversely related to muscle length, with shorter muscle lengths being more conducive to strength enhancement (Smith et al., 2011). The findings of this study align with those of Tsoukos et al. (2016), showing that the average power output of the 120° ISC was higher than that of both the CC and the 90° ISC. This could be due to the shorter muscle lengths involved in the 120° IS, allowing for greater phosphorylation of myosin light chains during CA compared to the 90° IS (Hamada et al., 2003; Tsoukos et al., 2016). This facilitated the faster recruitment of higher-order motor units, resulting in significantly greater average power output in the 120° ISC than in the CC and the 90° ISC. Nevertheless, neither the 90° nor the 120° ISC showed significant improvements in the F or P peaks during the SJ, with the F peak of the 90° ISC showing a slight decrease. Previous studies have suggested that changes in the mechanical properties of the muscle-tendon unit may shift the force-velocity curve to the right (Balnave and Allen, 1996), which could explain the phenomenon of enhanced velocity performance following the IS, while force performance remains unaffected.

A notable finding of this study is that ISs performed at different knee angles had varying effects on the SFV during the SJ at the corresponding angles. Both the 90° ISC and the 120° ISC demonstrated changes in the force-velocity relationship during their respective SJ, showing greater velocity components at the P peak compared to the CC. However, no significant changes were observed in SJs at non-corresponding angles. These differences may be attributed to the distinct effects of the 90° and 120° ISs on lower limb muscle activation patterns. The preliminary results indicated that the F peak at the 120° IS was higher than that at the 90° IS, which is consistent with previous research (Tan et al., 2024). This effect likely arises from changes in joint angles that alter the lever arms in skeletal motion, thereby influencing the force output (Millman, 1998; Worrell et al., 2001). As mentioned previously, RF activation levels were higher during the 90° IS than during the 120° IS, while SOL and TA activation were more pronounced during the 120° IS. According to the knee angle-torque relationship, the torque produced during isometric knee extension peaks at approximately 80° and then decreases (Marginson and Eston, 2001). Thus, the torque at a 120° knee angle is lower than that at 90°, which may explain the higher RF activation observed during the 90° IS. Moreover, smaller knee angles result in greater RF stretching, thereby providing stronger stimulation (Tsoukos et al., 2016). Muscle length during isometric contractions can also influence muscle fiber recruitment, thereby affecting the EMG amplitude (Christie et al., 2009; Worrell et al., 2001). These findings suggest that isometric deep squats at different knee angles can induce distinct activation patterns in lower limb muscles (Barrett et al., 2023). Based on the results of this study, it can be inferred that isometric contractions at specific muscle lengths effectively modify the force-velocity relationship under the initial contraction states, shifting the force-velocity curve to the right and increasing the velocity component at peak power. This finding has significant implications for athletes in velocity-dominant sports as it provides a basis for selecting appropriate knee angle IS protocols tailored to their individual characteristics and performance demands.

This study has certain limitations. First, it included only male participants, which may limit the applicability of the results to females. As previous research indicates that stronger individuals may experience greater effects of PAPE (Hamada et al., 2003; Tsoukos et al., 2016), we chose to exclude female participants to better focus on the effects of PAPE. Second, we only chose two angles for the isometric squat intervention, which may not be generalizable to all exercises. Future studies should explore a broader range of angles and intervention duration, such as lower knee angles or varying intervention lengths, for more detailed comparisons. Additionally, research suggests that combining ISs and augmented CA can effectively improve CMJ height (Kalinowski et al., 2022). A more nuanced understanding of the effects of ISs as CA on athletic performance could benefit a wide range of sports.

## Conclusions

Our results indicate that the 90° IS and the 120° IS elicited distinct stimulation of the lower limb muscles and had different effects on subsequent performance enhancement. For sports that require greater power output, the 120° IS as the CA may be a more suitable option than the 90° IS. However, both IS methods were found to increase velocity and effectively modify the force-velocity relationship at specific knee angles. This adjustment shifts the force-velocity curve to the right, thereby enhancing the velocity component at peak power. Therefore, coaches and athletes involved in high-speed sports should incorporate the IS at an appropriate knee angle into pre-competition or pre-training warm-up routines, tailored to individual training habits and specific performance demands.

## References

[ref1] Balnave, C., & Allen, D. (1996). The effect of muscle length on intracellular calcium and force in single fibres from mouse skeletal muscle. *Journal of Physiology*, 492(3), 705–713. 10.1113/jphysiol.1996.sp021339 8734983 PMC1158893

[ref2] Barbero, M., Merletti, R., & Rainoldi, A. (2012). Atlas of muscle innervation zones: understanding surface electromyography and its applications. Springer Science & Business Media. 10.1007/978-88-470-2463-2.

[ref3] Barrett, K. B., Sievert, Z. A., & Bennett, H. J. (2023). A comparison of squat depth and sex on knee kinematics and muscle activation. *Journal of Biomechanical Engineering*, 145(7), 071010. 10.1115/1.4062330 37066975

[ref4] Berning, J. M., Adams, K. J., DeBeliso, M., Sevene-Adams, P. G., Harris, C., & Stamford, B. A. (2010). Effect of functional isometric squats on vertical jump in trained and untrained men. *Journal of Strength & Conditioning Research*, 24(9), 2285–2289. 10.1519/JSC.0b013e3181e7ff9a 20683353

[ref5] Blazevich, A. J., & Babault, N. (2019). Post-activation potentiation versus post-activation performance enhancement in humans: Historical perspective, underlying mechanisms, and current issues. *Frontiers in Physiology*, 10, 1359. 10.3389/fphys.2019.01359 31736781 PMC6838751

[ref6] Bogdanis, G. C., Tsoukos, A., Veligekas, P., Tsolakis, C., & Terzis, G. (2014). Effects of muscle action type with equal impulse of conditioning activity on postactivation potentiation. *Journal of Strength and Conditioning Research*, 28(9), 2521–2528. 10.1519/JSC.0000000000000444 24584048

[ref7] Burden, A. J. J. o. e., & kinesiology. (2010). How should we normalize electromyograms obtained from healthy participants? What we have learned from over 25 years of research. *Journal of Electromyography and Kinesiology*, 20(6), 1023–1035. 10.1016/j.jelekin.2010.07.004 20702112

[ref8] Cady, E. B., Jones, D. A., Lynn, J., & Newham, D. J. (1989). Changes in force and intracellular metabolites during fatigue of human skeletal muscle. *Journal of Physiology*, 418, 311–325. 10.1113/jphysiol.1989.sp017842 2621621 PMC1189973

[ref9] Christie, A., Inglis, J. G., Kamen, G., & Gabriel, D. A. (2009). Relationships between surface EMG variables and motor unit firing rates. *European Journal of Applied Physiology*, 107(2), 177–185. 10.1007/s00421-009-1113-7 19544067

[ref10] Cohen, J. (1988). Statistical Power Analysis for the Behavioral Sciences (2nd ed.). Routledge. 10.4324/9780203771587

[ref11] Dobbs, W. C., Tolusso, D. V., Fedewa, M. V., & Esco, M. R. (2019). Effect of postactivation potentiation on explosive vertical jump: A systematic review and meta-analysis. *Journal of Strength and Conditioning Research*, 33(7), 2009–2018. 10.1519/JSC.0000000000002750 30138241

[ref12] Duchateau, J., & Hainaut, K. J. J. o. a. p. (1984). Isometric or dynamic training: differential effects on mechanical properties of a human muscle. *Journal of Applied Physiology*, 56(2), 296–301. 10.1152/jappl.1984.56.2.296 6706740

[ref13] Esformes, J. I., Bampouras, T. M. J. T. J. o. S., & Research, C. (2013). Effect of back squat depth on lower-body postactivation potentiation. *Journal of Strength and Conditioning Research*, 27(11), 2997–3000. 10.1519/JSC.0b013e31828d4465 23442291

[ref14] Fan, P., Kim, Y., Han, D.-W., Kim, S., & Wang, T. J. B. (2025). Alterations in the Neuromuscular Control Mechanism of the Legs During a Post-Fatigue Landing Make the Lower Limbs More Susceptible to Injury. *Bioengineering*, 12(3), 233. 10.3390/bioengineering12030233 40150697 PMC11939312

[ref15] Fan, P., Yang, Z., Wang, T., Li, J., Kim, Y., & Kim, S. J. (2024). Neuromuscular control strategies in basketball shooting: Distance-dependent analysis of muscle synergies. *Journal of Sports Science and Medicine*, 23(1), 571. 10.52082/jssm.2024.571 39228767 PMC11366846

[ref16] French, D. N., Kraemer, W. J., Cooke, C. B. J. T. J. o. S., & Research, C. (2003). Changes in dynamic exercise performance following a sequence of preconditioning isometric muscle actions. *Journal of Strength and Conditioning Research*, 17(4), 678–685. 10.1519/00124278-200311000-00009 14636092

[ref17] Gillen, Z. M., Shoemaker, M. E., McKay, B. D., Bohannon, N. A., Gibson, S. M., & Cramer, J. T. (2022). Influences of the stretch-shortening cycle and arm swing on vertical jump performance in children and adolescents. *Journal of Strength and Conditioning Research*, 36(5), 1245–1256. 10.1519/JSC.0000000000003647 32483060

[ref18] Hamada, T., Sale, D. G., MacDougall, J. D., & Tarnopolsky, M. A. (2003). Interaction of fibre type, potentiation and fatigue in human knee extensor muscles. *Acta Physiologica Scandinavica*, 178(2), 165–173. 10.1046/j.1365-201x.2003.01121.x 12780391

[ref19] Harland, M. J., & Steele, J. R. (1997). Biomechanics of the sprint start. *Sports Medicine*, 23(1), 11–20. 10.2165/00007256-199723010-00002 9017856

[ref20] Janićijević, D. N., Knežević, O. M., Mirkov, D. M., Pérez-Castilla, A., Petrović, M. R., & García-Ramos, A. (2019). Magnitude and reliability of mechanical outputs obtained during loaded squat jumps performed from different knee angles. *Sports Biomechanics*, 20(8), 925–937. 10.1080/14763141.2019.1618390 31232220

[ref21] Kalinowski, R., Pisz, A., Kolinger, D., Wilk, M., Stastny, P., & Krzysztofik, M. (2022). Acute effects of combined isometric and plyometric conditioning activities on sports performance and tendon stiffness in female volleyball players. *Frontiers in Physiology*, 13, 1025839. 10.3389/fphys.2022.1025839 36304585 PMC9593028

[ref22] Koźlenia, D., & Domaradzki, J. (2024). Postsubmaximal Isometric Full Squat Jump Potentiation in Trained Men. *Journal of Strength and Conditioning Research*, 38(3), 459–464. 10.1519/JSC.0000000000004647 37656774 PMC10880928

[ref23] Krishnan, C., & Williams, G. N. (2014). Effect of knee joint angle on side-to-side strength ratios. *Journal of Strength and Conditioning Research*, 28(10), 2981–2987. 10.1519/JSC.0000000000000476 24714535

[ref24] Krzysztofik, M., Spieszny, M., Trybulski, R., Wilk, M., Pisz, A., Kolinger, D., Filip-Stachnik, A., & Stastny, P. (2023). Acute effects of isometric conditioning activity on the viscoelastic properties of muscles and sprint and jumping performance in handball players. *Journal of Strength and Conditioning Research*, 37(7), 1486–1494. 10.1519/JSC.0000000000004404 36752742

[ref25] Laughlin, W. A., Weinhandl, J. T., Kernozek, T. W., Cobb, S. C., Keenan, K. G., & O'Connor, K. M. (2011). The effects of single-leg landing technique on ACL loading. *Journal of Biomechanics*, 44(10), 1845c1851. 10.1016/j.jbiomech.2011.04.010 21561623

[ref26] Li, M., Kim, Y., Guo, W., Fan, P., Wang, J., & Kim, S. (2024). Effects of conditioning contractions on lower-body explosive force post-static stretching. *International Journal of Sports Medicine*, 45(14), 1040–1046. 10.1055/a-2351-8735 38914131

[ref27] Li, M., Meng, X., Guan, L., Kim, Y., & Kim, S. (2023). Comparing the effects of static stretching alone and in combination with post-activation performance enhancement on squat jump performance at different knee starting angles. *Journal of Sports Science and Medicine*, 22, 769–777. 10.52082/jssm.2023.769 38045747 PMC10690507

[ref28] Loturco, I., Pereira, L. A., Moura, T. B., Mercer, V. P., Betelli, M. T., Ramos, M. S. … & Pareja-Blanco, F. (2024). Jump Squats Performed with Both Light and Heavy Loads Have Similar Effects on the Physical Performance of Elite Rugby Players during the Initial Phase of the Competitive Period. *Journal of Human Kinetics*, 91, 175–188. 10.5114/jhk/186340 38689591 PMC11057615

[ref29] Lum, D., Barbosa, T. M., Joseph, R., & Balasekaran, G. (2021). Effects of Two Isometric Strength Training Methods on Jump and Sprint Performances: A Randomized Controlled Trial. *Journal of Science in Sport and Exercise*, 3(2), 115–124. 10.1007/s42978-020-00095-w

[ref30] Lum, D., & Howatson, G. (2025). Comparing the acute effects of a session of isometric strength training with heavy resistance training on neuromuscular function. *Journal of Science in Sport and Exercise*, 7, 40–49. 10.1007/s42978-023-00241-0

[ref31] Marginson, V., & Eston, R. (2001). The relationship between torque and joint angle during knee extension in boys and men. *Journal of Sports Sciences*, 19(11–14), 875–880. 10.1080/026404101753113822 11695509

[ref32] McLellan, C. P., Lovell, D. I., & Gass, G. C. (2011). The role of rate of force development on vertical jump performance. *Journal of Strength and Conditioning Research*, 25(2), 379–385. 10.1519/JSC.0b013e3181be305c 20093963

[ref33] Millman, B. M. (1998). The filament lattice of striated muscle. *Physiological Reviews*, 78(2), 359–391. 10.1152/physrev.1998.78.2.359 9562033

[ref34] Mitchell, L. J., Argus, C. K., Taylor, K.-L., Sheppard, J. M., & Chapman, D. W. (2017). The effect of initial knee angle on concentric-only squat jump performance. Research Quarterly for Exercise and Sport, 88(2), 184–192. 10.1080/02701367.2017.1293777 28339350

[ref35] Samozino, P., Rejc, E., Di Prampero, P. E., Belli, A., & Morin, J.-B. (2012). Optimal force–velocity profile in ballistic movements—Altius: Citius or Fortius? *Medicine & Science in Sports & Exercise*, 44(2), 313–322. 10.1249/MSS.0b013e31822d757a 21775909

[ref36] Seitz, L. B., & Haff, G. G. (2016). Factors modulating post-activation potentiation of jump, sprint, throw, and upper-body ballistic performances: A systematic review with meta-analysis. *Sports Medicine (Auckland, N.Z.)*, 46(2), 231–240. 10.1007/s40279-015-0415-7 26508319

[ref37] Smith, C. B., Cheng, A. J., & Rice, C. L. (2011). Potentiation of the triceps brachii during voluntary submaximal contractions. *Muscle & Nerve*, 43(6), 859–865. 10.1002/mus.21993 21462211

[ref38] Stastny, P., Kolinger, D., Pisz, A., Wilk, M., Petruzela, J., & Krzysztofik, M. (2024). Effects of Eccentric Speed during Front Squat Conditioning Activity on Post-activation Performance Enhancement of Hip and Thigh Muscles. *Journal of Human Kinetics*, 91, 5–18. 10.5114/jhk/183917 38689578 PMC11057616

[ref39] Tan, W. Z. N., Lum, D. (2024). Predicting 1 Repetition Maximum Squat With Peak Force Obtained From Isometric Squat at Multiple Positions. *Journal of Strength and Conditioning Research*, 38(9), 1543–1550. 10.1519/JSC.0000000000004849 39074205

[ref40] Tillin, N. A., & Bishop, D. (2009). Factors modulating post-activation potentiation and its effect on performance of subsequent explosive activities. *Sports Medicine*, 39(2), 147–166. 10.2165/00007256-200939020-00004 19203135

[ref41] Tsoukos, A., Bogdanis, G. C., Terzis, G., & Veligekas, P. (2016). Acute improvement of vertical jump performance after isometric squats depends on knee angle and vertical jumping ability. *Journal of Strength and Conditioning Research*, 30(8), 2250–2257. 10.1519/JSC.0000000000001328 26808841

[ref42] Vargas-Molina, S., Salgado-Ramírez, U., Chulvi-Medrano, I., Carbone, L., Maroto-Izquierdo, S., & Benítez-Porres, J. (2021). Comparison of post-activation performance enhancement (PAPE) after isometric and isotonic exercise on vertical jump performance. *PLOS ONE*, 16(12), e0260866. 10.1371/journal.pone.0260866 34855891 PMC8639083

[ref43] Wilson, J. M., Duncan, N. M., Marin, P. J., Brown, L. E., Loenneke, J. P., Wilson, S. M., Jo, E., Lowery, R. P., & Ugrinowitsch, C. (2013). Meta-analysis of postactivation potentiation and power: Effects of conditioning activity, volume, gender, rest periods, and training status. *Journal of Strength and Conditioning Research*, 27(3), 854–859. 10.1519/JSC.0b013e31825c2bdb 22580978

[ref44] Worrell, T. W., Karst, G., Adamczyk, D., Moore, R., Stanley, C., Steimel, B., & Steimel, S. (2001). Influence of joint position on electromyographic and torque generation during maximal voluntary isometric contractions of the hamstrings and gluteus maximus muscles. *Journal of Orthopaedic and Sports Physical Therapy*, 31(12), 730–740. 10.2519/jospt.2001.31.12.730 11767248

[ref45] Zabaloy, S., Healy, R., Pereira, L. A., Tondelli, E., Tomaghelli, L., Aparicio, J. … & Loturco, I. (2025). A Randomized Controlled Trial of Unresisted vs. Heavy Resisted Sprint Training Programs: Effects on Strength, Jump, Unresisted and Resisted Sprint Performance in Youth Rugby Union Players. *Journal of Human Kinetics*, 95, 199–214. 10.5114/jhk/200121 39944984 PMC11812154

